# Effects of Dietary Chlorogenic Acid Supplementation Derived from *Lonicera macranthoides* Hand-Mazz on Growth Performance, Free Amino Acid Profile, and Muscle Protein Synthesis in a Finishing Pig Model

**DOI:** 10.1155/2022/6316611

**Published:** 2022-03-12

**Authors:** Wenlong Wang, Fengna Li, Yehui Duan, Qiuping Guo, Lingyu Zhang, Yuhuan Yang, Yunju Yin, Mengmeng Han, Saiming Gong, Jianzhong Li, Shanping He, Yulong Yin

**Affiliations:** ^1^National Engineering Laboratory for Pollution Control and Waste Utilization in Livestock and Poultry Production, Hunan Provincial Key Laboratory of Animal Nutritional Physiology and Metabolic Process, Key Laboratory of Agro-ecological Process in Subtropical Region, Institute of Subtropical Agriculture, Chinese Academy of Sciences, Changsha, Hunan 410125, China; ^2^Hunan Provincial Key Laboratory of Animal Intestinal Function and Regulation, Hunan International Joint Laboratory of Animal Intestinal Ecology and Health, Laboratory of Animal Nutrition and Human Health, School of Life Sciences, Hunan Normal University, Changsha, Hunan 410081, China; ^3^University of Chinese Academy of Sciences, Beijing 100049, China; ^4^College of Animal Science and Technology, Hunan Agricultural University, Changsha, Hunan 410128, China

## Abstract

Chlorogenic acid (CGA), as one of the richest polyphenol compounds in nature, has broad applications in many fields due to its various biological properties. However, initial data on the effects of dietary CGA on protein synthesis and related basal metabolic activity has rarely been reported. The current study is aimed at (1) determining whether dietary CGA supplementation improves the growth performance and carcass traits, (2) assessing whether dietary CGA alters the free amino acid profile, and (3) verifying whether dietary CGA promotes muscle protein synthesis in finishing pigs. Thirty-two (Large × White × Landrace) finishing barrows with an average initial body weight of 71.89 ± 0.92 kg were randomly allotted to 4 groups and fed diets supplemented with 0, 0.02%, 0.04%, and 0.08% CGA, respectively. The results indicated that, compared with the control group, dietary supplementation with 0.04% CGA slightly stimulated the growth performance of pigs, whereas no significant correlation was noted between the dietary CGA levels and animal growth (*P* > 0.05). Furthermore, the carcass traits of pigs were improved by 0.04% dietary CGA (*P* < 0.01). In addition, dietary CGA significantly improved the serum free amino acid profiles of pigs (*P* < 0.01), while 0.04% dietary CGA promoted more amino acids to translocate to skeletal muscles (*P* < 0.05). The relative mRNA expression levels of SNAT2 in both longissimus dorsi (LD) and biceps femoris (BF) muscles were augmented in the 0.02% and 0.04% groups (*P* < 0.05), and the LAT1 mRNA expression in the BF muscle was elevated in the 0.02% group (*P* < 0.05). We also found that dietary CGA supplementation at the levels of 0.04% or 0.08% promoted the expression of p-Akt and activated the mTOR-S6K1-4EBP1 axis in the LD muscle (*P* < 0.05). Besides, the MAFbx mRNA abundance in the 0.02% and 0.04% groups was significantly lower (*P* < 0.05). Our results revealed that dietary supplementation with CGA of 0.04% improved the free amino acid profile and enhanced muscle protein biosynthesis in the LD muscle in finishing pigs.

## 1. Introduction

Studies on natural substances and herbal plant extracts have become increasingly popular due to their numerous beneficial properties. As one of the most widespread plant extracts in nature, chlorogenic acid (CGA) has received growing attention [1–3]. It has been verified to display multiple biological and pharmacological properties, including antioxidative [4–7], anti-inflammatory [5, 8–11], antibacterial [12, 13], and antiviral [14, 15] activities. Subsequently, its potential role in glucose and lipid metabolism has been explored [16, 17]. CGA has been found to have the potential to moderate improvements in glycemic and blood pressure control [18, 19]. There is also solid evidence that CGA is an adequate glucose and lipid metabolism regulation agent [20, 21]. These properties have a broad range of applications in human health care [7, 22], animal production, and the food industry [23, 24].

In addition, CGA is extensively distributed in nature and can be detected in kinds of foods and herbs, such as coffee beans, apples, pears, potatoes, and tea [25, 26]. Among them, *Flos lonicerae* (*F. lonicerae*) and *Eucommia ulmoides* (*E. ulmoide*) are the two crucial CGA resources. In China, especially in Hunan Province, they are widely cultivated [27, 28]. The Longhui county and Zhangjiajie districts in Hunan Province are two main producing areas of *Lonicera macranthoides* (one of *F. lonicerae* species; *L. macranthoides*) and *E. ulmoide*, respectively [27, 28]. So far, they have been widely used in medicine, tea, and food [28]. However, it was found that some low-quality products and CGA-rich extract residuals were abandoned and caused specific serious problems, such as the waste of natural resources and environmental pollution [28]. It can be a conductive solution to use these underutilized parts of natural resources as animal feed supplements [28]. Hence, it is of great significance to reveal the action mechanism of CGA, tap into the potential of local resources, and promote local economic development [28].

Theoretically, regardless of the application area, initial data on the effects of dietary CGA on the basal physiological process is indispensable. In particular, protein synthesis, considered one of the most important physiological activities, should be scrutinized. However, only a handful of relevant studies have been published. In reviewing these studies, we found that supplementation with polyphenols had been demonstrated to enhance recovery from muscle damage induced by intensive exercise or high-fat diet-induced muscle atrophy due to the involvement of inflammation damage, oxidative stress, and mitochondrial dysfunction within muscles [29]. A recent report [30] indicated that treatment using pharmacological ingredients (polyphenols, including curcumin and resveratrol) known to promote sirtuin-1 activity might enhance muscle regeneration. Another assay [31] on patients with chronic kidney disease and iron overload demonstrated the beneficial effects of combined supplementation with curcumin and resveratrol on muscle and bone mass. In particular, published studies have described the beneficial effect of each polyphenol subclass on muscle mass preservation in various skeletal muscle disorders [32]. Furthermore, a separate review highlighted recent evidence, most of which were carried out in mice, for the ability of polyphenols and their derivatives to reduce muscle wasting in different pathological states [33]. Moreover, polyphenols have also been reported to improve skeletal muscle function [34], promote protein synthesis [35], and increase muscle weight [36].

Given the current information on polyphenols, we hypothesized that CGA might potentially elevate the free amino acid profile, enhance muscle protein synthesis and, therefore, improve muscle mass. Nevertheless, few studies have focused on the effects of CGA on free amino acid profile, protein synthesis, and muscle mass, let alone using a finishing pig model. In fact, in addition to acting as the most common meat provider, pigs are also an ideal animal model for studying human obesity, diabetes, and metabolic syndrome due to the similarity in anatomic and physiologic characteristics [37–39]. Therefore, this study is aimed at investigating the mechanism responsible for the effects of dietary CGA supplementation on muscle protein synthesis. To clarify this phenomenon, we mainly focused on whether dietary CGA promotes growth performance, the free amino acid profile, and Akt-mTOR-S6K1-4EBP1 signaling components involved in protein synthesis in the skeletal muscles.

## 2. Materials and Methods

### 2.1. Animals and Experimental Design

The experimental design and all animal procedures were approved by the Animal Care Committee of the Institute of Subtropical Agriculture, Chinese Academy of Sciences (The ethical approval code is a20160606, Changsha, China). Thirty-two (Large × White × Landrace) barrows, with an initial body weight (BW) of 71.89 ± 0.92 kg, were selected and fed a corn and soybean meal-based diet according to the recommendation of the National Research Council [40] for five days. They were then randomly assigned to four groups with eight pigs (replicates) in each group and fed with a basal diet supplemented with 0, 0.02%, 0.04%, or 0.08% of CGA (control group, 0.02% group, 0.04% group, and 0.08% group; Table S1). CGA was obtained from Changsha E. K Herb Co., Ltd. in terms of a purified extract product (>98%) derived from *L. macranthoides* in Longhui County, Hunan Province. All pigs were used for experimental purposes only. Each pig was randomly housed in a single pen (2.2 m∗1.0 m) to ensure stand and avoid fights and move freely. During the 35-day experimental period, all pigs were provided ad libitum access to the diets and clean drinking water. The feed intake was recorded every seven days to determine the average daily feed intake (ADFI), average daily gain (ADG), and feed intake to body gain ratio (F/G). All pigs were weighed at the beginning and end of the trial.

### 2.2. Sample Collection

At the end of the feeding trial, blood samples from the overnight fasting (12 h) pigs were collected into 10 mL tubes by inferior vena cava puncture to evaluate serum biochemical parameters. All blood samples were allowed to clot at room temperature. The serum was separated immediately by centrifugation (3,000 × *g*, 15 min, 4°C) and then stored at –80°C until further use. The pigs were slaughtered via electrical stunning (250 V, 0.5 A, 5–6 s) and then exsanguinated and eviscerated. Subsequently, the carcass was weighed and longitudinally split. The longissimus dorsi (LD) and biceps femoris (BF) muscle samples were rapidly excised from the right side of the carcass. Samples from the LD and BF muscles were immediately frozen in liquid nitrogen and then stored at –80°C until further analysis.

### 2.3. Determination of Carcass Traits

The left side of the carcass was weighed, and the carcass length and fat thickness were measured. The carcass length was denoted as the distance from the upper margin of the symphysis pubis to the junction of the first rib and the sternum. The fat thickness was measured between the sixth and seventh ribs. Then, the left side of the carcass was dissected into the skeletal muscle, fat, bone, and skin. The carcass composition was calculated by dividing the dissected tissues by carcass weight of the left side. The dressing percentage was calculated as carcass weight divided by the slaughter weight ×100%. Next, the left side of the carcass was split at the tenth rib to determine the longissimus dorsi muscle area (LMA). The fat-free lean percentage and body fat rate were calculated according to the equations suggested by the National Pork Producers Council [41].

### 2.4. Measurement of Serum Metabolites

Serum biochemical parameters, including total protein (TP), albumin (ALB), globulin (GLB), urea (UR), and creatinine (CREA), were determined using a Roche automatic biochemical analyzer (Cobas c311; F. Hoffmann-La Roche Ltd., Basel, Switzerland) with relevant commercially available kits (F. Hoffmann-La Roche Ltd., Basel, Switzerland).

### 2.5. Evaluation of Free Amino Acid Profile

The serum free amino acid profile was determined using a modified high-performance liquid chromatography method based on previous studies [42, 43]. Briefly, the serum was centrifuged at 3,000 × *g* for 10 min. The supernatant (approximately 1 mL) was thoroughly homogenized with the same volume of 0.8% sulfosalicylic acid solution. After incubating at room temperature for 15 min, the mixture was centrifuged at 10,000 × *g* for 10 min. The supernatant was then homogenized with 2 mL of n-hexane solution. After demixing, the subnatant was filtered through a 0.22 mm microfilter. The filtrate was labeled with iTRAQ reagents (AA 45/32 kit; Applied Biosystems, Foster City, CA, USA) as recommended by the manufacturer and analyzed on a high-performance liquid chromatograph (1260 Infinity II; Agilent, Santa Clara, CA, USA).

The free amino acid profile was determined in the LD and BF muscles using a modified high-performance liquid chromatography method based on previous studies [42, 43]. Approximately 2.5 g fresh meat sample was thoroughly homogenized with 10 mL of a 0.01 M hydrochloric acid solution. The mixture was stirred in an ultrasonic bath for 30 min. Then, the supernatant was transferred to a 25 mL volumetric flask, diluted with distilled water, and filtered. The filtrate (2 mL) was homogenized with an equal volume of n-hexane solution. After demixing, the subnatant (1 mL) was thoroughly homogenized with the same volume of 0.8% sulfosalicylic acid solution and incubated at 4°C overnight. The mixture was centrifuged at 10,000 × *g* for 10 min. Subsequently, the supernatant was filtered through a 0.22 mm microfilter. Finally, the filtrate was labeled with iTRAQ reagents (AA 45/32 kit; Applied Biosystems, Foster City, CA, USA) as recommended by the manufacturer and analyzed using the same high-performance liquid chromatography method described above.

### 2.6. Real-Time PCR

The isolation of total RNA and determination of the quality and quantity of RNA were according to the methods we described before [44]. The primer sequences of the selected genes are listed in Table S2.

### 2.7. Western Blotting

Western blot analysis was performed using standard protocols. Briefly, approximately 30 *μ*g of total protein extracted from muscle samples were separated using SDS-PAGE and transferred onto polyvinylidene fluoride (PVDF) membranes (Millipore, Billerica, MA, USA). After being blocked with 5% nonfat milk, the membranes were incubated with primary antibodies (Cell Signaling Technology, Beverly, MA, USA) overnight at 4°C at a dilution of 1 : 1000. The membranes were then washed in TBST buffer and incubated with secondary antibody peroxidase-conjugated anti-goat/anti-rabbit IgG (Santa Cruz Biotechnology, Santa Cruz, CA, USA) for 1 h at a dilution of 1 : 5000. The bands were visualized with a chemiluminescent reagent (Pierce, Rockford, IL, USA) on a ChemiDoc XRS Gel Imaging System (Bio-Rad, Hercules, CA, USA). Quantity One 1-D analysis software (version 4.6.2; Bio-Rad, Hercules, CA, USA) was used to quantify the resultant signals. The primary antibodies used were as follows: Phospho-mTOR (Ser2448; #5536, CST), mTOR (#2972, CST), Phospho-4EBP1 (Ser65; #9451, CST), 4EBP1 (#9452, CST), Phospho-p70S6 kinase (Thr389; #9205, CST), p70S6 kinase (#9202, CST), Phospho-Akt (#11962, CST), and *β*-tubulin (#2128, CST).

### 2.8. Statistical Analysis

Data were analyzed for significance using the SAS 8.2 software (SAS Institute Inc., Cary, NC, USA) by one-way ANOVA followed by Duncan's multiple comparisons. Mean differences between groups were considered statistically significant at *P* < 0.05 and displayed a tendency towards significance at 0.05 < *P* < 0.10. Graphs were prepared using the Graphpad Prism 7.0 software (GraphPad Software Inc., San Diego, CA, USA).

## 3. Results

### 3.1. Growth Performance

The results obtained from the growth performance analysis of finishing pigs fed with various CGA supplementation levels are provided in Table 1. No significant differences were recorded in the initial weight, final weight, and ADFI between the four groups (*P* > 0.05). Furthermore, compared with the control group, the ADG and F/G of the pigs in the diet treatment groups were not observably altered (*P* > 0.05). However, further analysis showed that the ADG of the pigs in the 0.04% group was the maximum value, and it was significantly improved compared to that in the 0.02% group (*P* < 0.05). Similarly, the 0.04% group showed the lowest F/G value, which was decreased by 22.85% compared to that in the 0.08% group (*P* < 0.05). Thus, the results in this chapter indicate that dietary supplementation with CGA at a level of 0.04% slightly stimulated the growth performance of pigs, while no significant correlation was noted between dietary CGA and animal growth (*P* > 0.05).

### 3.2. Carcass Traits

The carcass traits of pigs in the different diet groups are summarized in Table 2. The fat-free lean percentage of pigs in the 0.04% group was markedly higher than in the other groups (*P* < 0.01). Compared with the 0.08% group, the fat percentage of the 0.02% and 0.04% groups was reduced by 21.62% and 26.49%, respectively (*P* < 0.01). In addition, the minimum backfat thickness found in the 0.04% group was dramatically diminished compared to the 0.08% group (*P* < 0.05) and displayed a decreasing tendency compared with the control group (0.05 < *P* < 0.10). The most disappointing result to emerge from the data was the lack of a correlation between dietary CGA and the LMA of pigs (*P* > 0.05). Besides, no significant differences were detected in slaughter weight, carcass weight, dressing percentage, or carcass length between the four groups (*P* > 0.05). Together, these results provide important insights into how dietary supplementation with 0.04% CGA increases the fat-free lean percentage and decreases fat deposition without adverse effects on the pigs' slaughter weight.

### 3.3. Serum Metabolites

The serum metabolites levels of finishing pigs fed diets supplemented with CGA are presented in Table 3. Compared with the 0.08% group, the TP content of the 0.02% and 0.04% groups was significantly decreased (*P* < 0.01), and no significant differences were noted between the control and CGA supplementation groups (*P* > 0.05). The ALB level of the 0.08% group was the highest, which was 15.23% higher than that of the 0.04% group (*P* > 0.05). Similarly, the serum GLB level of the 0.08% group was distinctly higher than that of the 0.02% and 0.04% groups (*P* < 0.01). Regarding the level of serum UR, dietary CGA significantly decreased the serum UR level compared to the control group (*P* < 0.01), and no statistical difference was found between the CGA supplementation groups (*P* > 0.05). Moreover, the serum CREA levels of the pigs were not significantly influenced by dietary CGA (*P* > 0.05). Nevertheless, the serum CREA content of the 0.04% group was noticeably higher than that of the 0.02% group (*P* < 0.05).

### 3.4. Free Amino Acid Profile

As shown in Table 4, compared with the control group, the serum EAA and NEAA levels of the CGA supplementation groups were significantly increased (*P* < 0.01). Other than Ala and NEAAs, all data between the CGA supplementation groups were similar. Regarding Ala and NEAAs, the data of the 0.08% group was higher than that of the 0.04% group (*P* < 0.01). These results imply that dietary CGA supplementation markedly increases the serum amino acid profile to provide more amino acids for protein synthesis.

We also evaluated the free amino acid levels in skeletal muscles to determine whether the increased free amino acids in the serum were translocated to the muscles. Tables 5 and 6 compare the free amino acid profiles in the LD and BF muscles, respectively. In the LD muscle, the Lys level of the 0.04% group was significantly higher than those of the other groups (*P* < 0.01). Likewise, the Val levels of the 0.04% group were prominently enhanced in contrast to the control and 0.02% groups (*P* < 0.05). Regarding NEAAs, the Cys and Tyr levels of the 0.02% and 0.04% groups were sharply increased compared to the control group (*P* < 0.05), and the Cys and Tyr levels of the 0.08% group displayed an increased tendency compared with the control (0.05 < *P* < 0.10). However, the effects of dietary CGA on BF muscles were somewhat different. In the BF muscle, the Ile content of the 0.04% group was 25.56% lower than the 0.08% group (*P* < 0.05), while it was similar to the control and 0.02% groups (*P* > 0.05). Compared with the other groups, dietary supplementation with CGA at a level of 0.04% substantially boosted the Thr content of the 0.04% group (*P* < 0.01). As shown in Table 6, no differences were found in the BCAA levels between the control and CGA supplementation groups (*P* > 0.05), while the BCAA levels of the 0.02% and 0.04% groups were statistically lower than that of the 0.08% group (*P* < 0.05). In contrast to the control, the 0.02% group showed a significantly reduced Asp level and a distinctly improved Gly content (*P* < 0.05). Furthermore, the Gly content of the 0.02% group was also significantly higher than that of the 0.04% group (*P* < 0.05). The 0.08% group exhibited decreased Ser and Pro levels, which were obviously lower than those of the other groups (*P* < 0.01). In addition, the Ser in BF muscles of the pigs in the 0.04% group was significantly higher than those of the control and 0.02% groups (*P* < 0.01). In summary, these results suggest that dietary supplementation with 0.04% CGA promotes the translocation of amino acids to skeletal muscles to participate in protein synthesis.

### 3.5. Relative mRNA Expression Levels of Major Amino Acid Transporters in Skeletal Muscles

**Figure 1:**
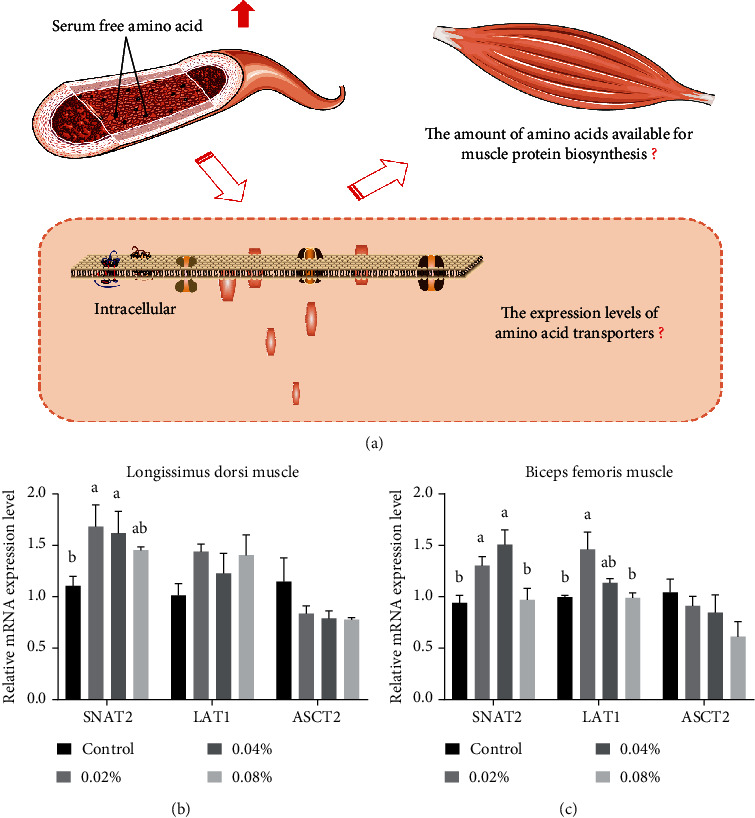
Effects of different levels of dietary CGA supplementation on relative mRNA expression levels of essential amino acid transporters in skeletal muscles of finishing pigs. (a) The amino acids available for protein biosynthesis in skeletal muscles rely on the uptake and translocation by amino acid transporters. (b) Relative mRNA expression levels of primary amino acid transporters in LD muscles. (c) Relative mRNA expression levels of primary amino acid transporters in BF muscles. Values are presented as the mean values, with their standard errors represented by vertical bars. ^a,b,c^Values in the same row with different superscript letters are significantly different at *P* < 0.05.

Real-time PCR analysis was used to determine the effect of dietary CGA supplementation on the relative mRNA expression levels of major amino acid transporters in the skeletal muscles of finishing pigs. The results are outlined in Figures 1(b) and 1(c). In the LD muscle, compared with the control group, the SNAT2 mRNA expression of the 0.02% and 0.04% groups was conspicuously enhanced (*P* < 0.05), and the 0.08% group displayed an increasing tendency (0.05 < *P* < 0.10). Surprisingly, the mRNA expression of LAT1 and ASCT2 was completely unaffected by dietary CGA supplementation (*P* > 0.05). Unlike the LD muscle, the mRNA expression of SNAT2 and LAT1 was modulated by dietary CGA in the BF muscle. The SNAT2 mRNA abundance of the 0.02% and 0.04% groups was observably higher than that of the control (*P* < 0.05). In terms of the LAT1 mRNA abundance, the 0.02% group was noticeably richer than the control group, and the 0.04% group showed a trend to intensify its expression (0.05 < *P* < 0.10). This result indicates that dietary CGA strengthens the mRNA expression of major amino acid transporters, such as SNAT2 and LAT1, in the skeletal muscles of finishing pigs.

### 3.6. Relative Abundance of Key Proteins Related to Muscle Protein Synthesis

**Figure 2:**
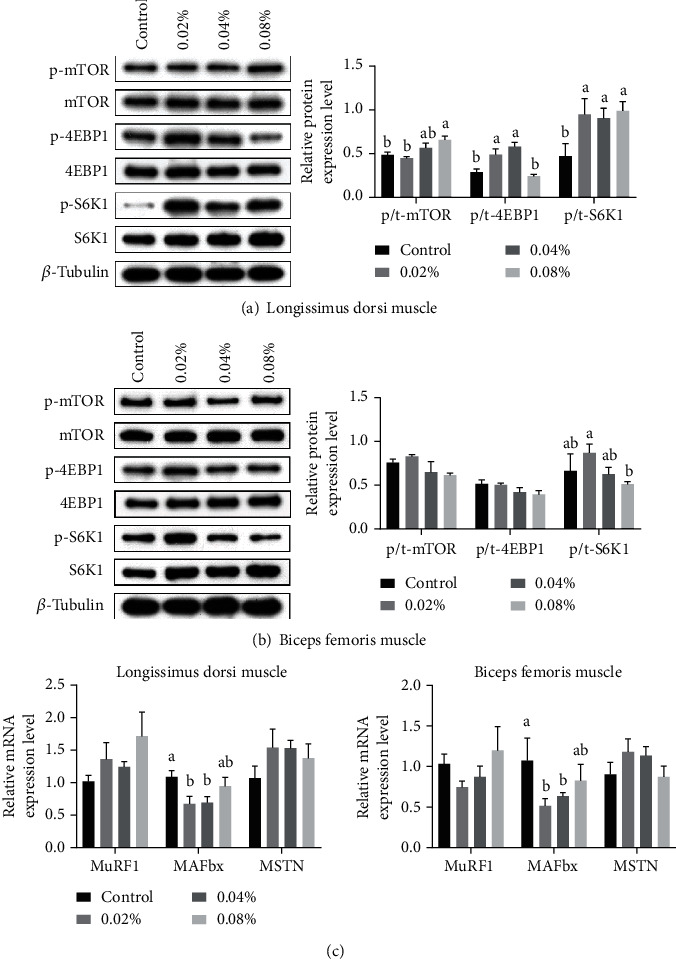
Effects of different levels of dietary CGA supplementation on muscle protein synthesis in the skeletal muscles of finishing pigs. (a) Relative abundance of momentous proteins related to protein synthesis in LD muscles. (b) Relative abundance of momentous proteins related to protein synthesis in BF muscles. (c) Relative mRNA expression levels of skeletal muscle protein degradation-related genes. Values are presented as the mean values, with their standard errors represented by vertical bars. p/t denotes the corresponding phosphorylated protein normalized to the total protein level. ^a,b,c^Values in the same row with different superscript letters are significantly different at *P* < 0.05.

The effects of dietary CGA supplementation on the relative abundance of key proteins related to protein synthesis in the skeletal muscles of finishing pigs were evaluated by western blotting. The results of the correlational analysis are set out in Figure 2. A positive correlation was found between dietary CGA and protein phosphorylation of mTOR, 4EBP1, and S6K1 in the LD muscle (Figure 2(a)). Compared with the control group, the protein phosphorylation of S6K1 in the diet treatment groups was significantly activated (*P* < 0.05). Interestingly, a statistical difference in the protein phosphorylation of 4EBP1 between the control and 0.02% or 0.04% groups was observed (*P* < 0.05). Consequently, the p/t-mTOR level of the 0.08% group was significantly increased (*P* < 0.05), while the 0.04% group showed an increasing trend (0.05 < *P* < 0.10). As shown in Figure 2(b), in the BF muscle, the protein phosphorylation levels of mTOR, 4EBP1, and S6K1 were unaffected by dietary CGA (*P* > 0.05). In addition, regarding the relative expression level of p/t-S6K1, the 0.02% group tended to elevate while the 0.08% group tended to reduce (0.05 < *P* < 0.10). Overall, dietary supplementation with 0.02% and 0.04% CGA enabled the activation of the mTOR-S6K1-4EBP1 axis in the LD muscle and had little effect on the BF muscle.

The relative mRNA expression levels of skeletal muscle protein degradation-related genes were assessed by real-time PCR analysis. As shown in Figure 2(c), the relative mRNA expression levels of MuRF1 and MSTN in both the LD and BF muscles were unaffected (*P* > 0.05). However, the MAFbx mRNA abundance of the 0.02% and 0.04% groups was prominently lower (*P* < 0.05). Meanwhile, a decreased tendency in the MAFbx mRNA expression was noted between the control and 0.08% groups (0.05 < *P* < 0.10). These results suggest that dietary CGA supplementation (especially at the level of 0.02% or 0.04%) promotes muscle protein synthesis by downregulating the mRNA expression of the protein degradation-related gene MAFbx.

**Figure 3:**
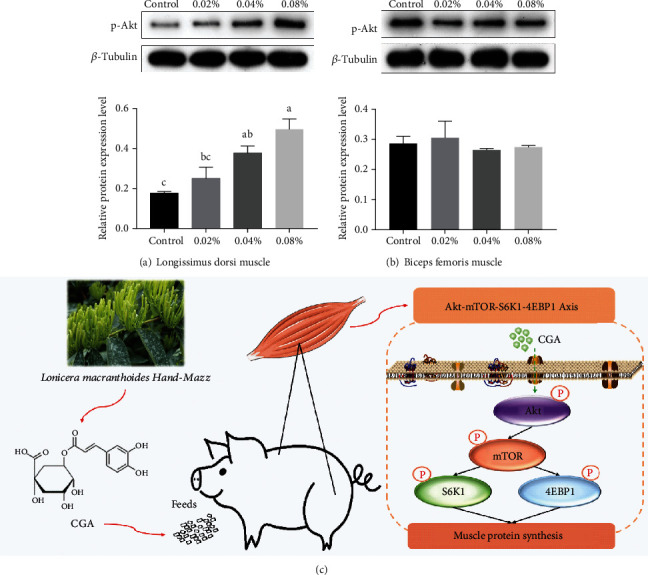
Effects of different levels of dietary CGA supplementation on the upper pathway of muscle protein synthesis. (a) Relative abundance of protein p-Akt in LD muscles. (b) Relative abundance of protein p-Akt in BF muscles. (c) The action mechanism of dietary CGA on skeletal muscle protein synthesis. Values are presented as the mean values, with their standard errors represented by vertical bars. ^a,b,c^Values in the same row with different superscript letters are significantly different at *P* < 0.05.

Moreover, the protein abundance in the upper pathway of muscle protein synthesis was assessed. As shown in Figures 3(a) and 3(b), dietary CGA supplementation increasingly activated p-Akt in the LD muscle (*P* < 0.05), while dietary CGA had no significant effect on p-Akt in the BF muscle (*P* > 0.05). The protein abundance of p-Akt in the LD muscle of pigs in the 0.04% and 0.08% groups was apparently enhanced than the control group (*P* < 0.05). Compared with the 0.02% group, the protein abundance of p-Akt in the LD muscle of pigs in the 0.08% groups was signally higher (*P* < 0.05). However, there was no significant difference between the protein abundance of p-Akt in the LD muscle of pigs in the 0.04% and 0.08% groups (*P* > 0.05). The result we collected was in agreement with the evaluation of the relative abundance of key proteins related to protein synthesis in the skeletal muscles. It indicated that dietary CGA supplementation may active the mTOR-4EBP1-S6K1 axis in skeletal muscles by enhancing the upper key protein p-Akt (as shown in Figure 3(c)). Thus, the clue of this paper was clear to see that dietary CGA enhanced the expression of p-Akt, inducing the activation of the mTOR-4EBP1-S6K1 axis in the LD muscle, while CGA influenced little on the BF muscle.

## 4. Discussion

A growing body of evidence has noted the benefits of CGA on the health and development of human beings and animals [3, 17]. However, in reviewing prior literature, few studies have reported the effects of CGA on protein synthesis, even though protein synthesis is a fundamental physiological process. Herein, our data demonstrated that a substantial amount (especially at the level of 0.04%) of CGA intake slightly stimulated the growth performance, while the 0.08% group showed a significantly decreased F/G value compared with the 0.04% group. Besides, dietary supplementation with 0.04% CGA was conducive to increasing fat-free lean percentage and decreasing fat deposition without adverse effects on the pigs' slaughter weight. Overall, 0.04% dietary CGA supplementation results in a better improvement than 0.02% or 0.08%. This positive result provides a foundation for follow-up research and the application of CGA. Furthermore, in the present study, dietary CGA strengthens the mRNA expression of major amino acid transporters. Also, dietary CGA supplementation (especially at the level of 0.02% or 0.04%) downregulates the mRNA expression of the protein degradation-related gene MAFbx. Moreover, further analysis reveals the potential of dietary CGA to promote protein synthesis via the Akt-mTOR-S6K1-4EBP1 signaling pathway in the LD muscle. Interestingly, these results, which identified with the former report, manifested that oversupplementation of CGA (0.08%) may not enlarge even reverse the beneficial effects [28] and thus led to the lack of progressivity (0.02%-0.04%-0.08%) in the beneficial effects of dietary CGA. The insights gained from this study provide essential data for the development and utilization of CGA in human and animal health.

Blood biochemical parameters are considered the most sensitive indicators of the response to nutrients and stress. As the leading and ultimate nitrogenous product of protein catabolism, serum urea (UR) acts as an index of protein utilization efficiency and reflects the balance status of amino acids [45]. Considerable studies have manifested that serum UR reduction presumably reflects a more efficient total nitrogen (N) utilization and an improved N balance [46–48]. We speculated that the underlying mechanism is likely to be associated with excess dietary amino acids that cannot be stored and instead produce UR [45]. It has also been well documented that increasing amino acid utilization decreases UR synthesis and the serum UR concentration [48]. The results herein suggest that dietary CGA supplementation may improve feed N utilization, leading to decreased serum UR content. Moreover, creatinine (CREA) is closely related to muscle production as a degradation product of creatinine in muscle cells [49]. It has been proved that high muscle production alone is correlated with elevated serum creatinine [50]. Coincidentally, in this study, the serum CREA content of pigs fed with CGA at a level of 0.04% exhibited an increasing tendency compared to the control, highlighting the potential of dietary CGA to lower the serum CREA content. This result is consistent with previous reports. Taken together, we conclude that dietary supplementation with 0.04% CGA contributes to improving fat-free lean mass. Furthermore, our data on the relative expression levels of protein metabolism-related proteins and mRNAs support this conclusion.

Interestingly, in this study, the content of serum free amino acids in pigs fed with CGA supplementation increased sharply compared with the control. This result was in accordance with the improvement in total N utilization triggered by dietary CGA. Besides, it is well known that free amino acids are not only the basic building blocks for tissue protein synthesis but also the prerequisite substrates needed to synthesize nitrogenous substances with great physiological importance [51, 52]. From this perspective, the free amino acid concentration in tissues reflects the nutritional state and physiological conditions (including protein metabolism) in animals to a certain degree [53–55]. Furthermore, the protein synthesis process has been reported to be regulated by the intracellular presence of available amino acids [56]. In skeletal muscles, where the most considerable free amino acid pool is stored inside the body, free amino acids are essential for growth by regulating protein turnover [57–59]. Therefore, if we assume that the flux of free amino acids in skeletal muscles increases along with the serum free amino acid concentration, it can be speculated that protein synthesis is enhanced. Our results showed that dietary CGA supplementation induced the increase in free amino acid concentration in the blood and as well as the muscles, particularly the free amino acid levels of the 0.04% group. Thus, we speculate that dietary CGA enables the enhancement of muscle protein synthesis. However, further analyses are required to confirm this inference. Since free amino acids impact muscle protein deposition after being utilized by skeletal muscles and the core process of the absorption of free amino acids is mainly mediated by specific transporters, it is necessary to identify the changes in the expression of these amino acid transporters (Figure 1(a)) [51, 60].

Amino acid transporters, ubiquitously distributed in the membranes of many different cell types, act as gatekeepers for muscle cells, playing a vital role in sensing the availability of amino acids, relaying nutrient signals to the cell interior, and controlling the uptake and efflux of amino acids [51, 61, 62]. Namely, the intracellular presence of available amino acids is modulated by the coordinated activity of amino acid transporters (Figure 1(a)) [45, 56, 60, 61]. Emerging evidence indicates that amino acid transporters exhibit a dual transporter/receptor (transceptor) function [61, 62]. These transceptors may modulate nutrient signaling, such as the mammalian target of rapamycin (mTOR) signaling pathway, via their ability to sense changes in the intracellular free amino acid pool and extracellular amino acids [51, 62]. Among these transceptors, SNAT2 (sodium-coupled neutral amino acid transporter 2; system A transporter, also known as SLC38A2) [63], LAT1 (L-type amino acid transporter 1; also known as SLC7A5) [64], and ASCT2 (alanine-serine-cysteine transporter 2; also known as SLC1A5) have been reported to exhibit the characteristics of transceptors and participate in the activation of the target of rapamycin protein complex [65–67]. Our data show that dietary CGA (at levels of 0.02% and 0.04%) strengthens the mRNA expression of muscle amino acid transporters, such as SNAT2 and LAT1. This part conforms with the corresponding results for the concentrations of free amino acids in skeletal muscles. It also implies that dietary CGA additives may play a role in readjusting the mTOR signaling pathway and thus the protein synthesis process in skeletal muscles.

mTOR is a crucial signaling node in regulating diverse aspects of cellular physiology such as body metabolism, protein synthesis, cell size, autophagy, cell growth, and differentiation [68–70]. mTOR kinase acts in two functionally distinct complexes, mTOR complex 1 (mTORC1) and 2 (mTORC2), whose activities and substrate specificities are regulated by complex cofactors [70]. One of the best-studied nutrient signaling cascades in higher eukaryotes is the mammalian target of the mTORC1 pathway, which plays a decisive role in controlling protein synthesis [71]. The activation of mTORC1 most prominently results in the phosphorylation of two downstream targets, p70 ribosomal S6 kinase protein (S6K1) and eukaryotic translation initiation factor 4E-binding protein 1 (4EBP1), which stimulate ribosome biogenesis and protein synthesis [69, 72]. Hence, the mTOR-S6K1-4EBP1 signaling pathway is deemed to be involved in the molecular mechanisms responsible for regulating protein synthesis. In the current study, a substantial amount of dietary CGA supplementation (at levels of 0.04% and 0.08%) was found to promote the protein phosphorylation of p-Akt and then motivate the mTOR-S6K1-4EBP1 signaling pathway in the LD muscle. However, comparatively, minor effects were observed in the Akt-mTOR-S6K1-4EBP1 axis in the BF muscle. The discrepancy regarding the effects of dietary CGA supplementation on the LD and BF muscles was ascribed to their different proportions of oxidative and glycolytic myofibers [73], which resulted in a big difference in the degree of fatty acid oxidation, ATP production, and protein synthesis progress. In addition, skeletal muscle mass, a reflection of protein accretion, is predicated upon the balance between protein synthesis and degradation [74]. Therefore, we examined the gene expression of muscle-specific ubiquitin ligases, namely, muscle atrophy F-box (MAFbx), muscle-specific RING-finger protein 1 (MuRF1), and myostatin (MSTN), to evaluate the status of muscle protein degradation. Unexpectedly, the results showed that 0.04% and 0.08% dietary CGA significantly downregulated the expression of MAFbx.

To date, the relationship between dietary CGA supplementation and protein metabolism has rarely been reported. This study attempted to verify whether dietary CGA affected amino acid profile and muscle protein synthesis in pigs. Unfortunately, some limitations in the current study should be concerned: (1) a lack of direct data from the basal metabolic test to evaluate the N utilization efficiency and N balance and (2) ambiguous action mechanism of dietary CGA on protein degradation. Due to the complicated molecular dynamics, further studies are needed to identify the role of CGA in regulating N utilization efficiency and protein degradation pathway to promote the development and utilization of CGA.

## 5. Conclusions

In conclusion, this study demonstrates that dietary supplementation with 0.04% CGA in finishing pigs improves the free amino acid profile and enhances protein biosynthesis in the LD muscle via the Akt-mTOR-S6K1-4EBP1 axis. Despite its exploratory nature, this study provides some insights into the improvement of natural plant extracts in energy conservation, waste product utilization, and pollution reduction. It also provides a practical reference for therapeutic strategies targeting skeletal muscle-related diseases, including muscular dystrophy and sarcopenia. Thus, these findings have critical implications for the food industry, animal production practice, and human health care in the future.

## Figures and Tables

**Table 1:** Growth performance of finishing pigs with different levels of dietary CGA supplementation.

Items	Control	0.02%	0.04%	0.08%	SEM	*P* value
Initial weight (kg)	72.40	71.67	71.92	71.58	0.92	0.99
Final weight (kg)	96.80	95.15	96.70	92.92	1.14	0.62
ADG^1^ (g/d)	750.55^ab^	688.71^b^	844.52^a^	725.16^ab^	25.50	0.07
ADFI^2^ (g/d)	2446.00	2225.20	2264.00	2400.90	58.57	0.53
F/G^3^	3.36^ab^	3.27^ab^	2.87^b^	3.72^a^	0.14	0.06

^1^ADG: average daily gain. ^2^ADFI: average daily feed intake. ^3^F/G: feed intake to body weight gain ratio. ^a,b,c^Values in the same row with different superscript letters are significantly different at *P* < 0.05.

**Table 2:** Carcass traits of finishing pigs with different levels of dietary CGA supplementation.

Items	Control	0.02%	0.04%	0.08%	SEM	*P* value
Slaughter weight (kg)	96.80	95.15	96.70	92.92	1.41	0.62
Carcass weight (kg)	65.50	65.32	66.90	65.28	1.48	0.98
Dressing percentage (%)	70.41	70.92	70.49	71.90	1.20	0.42
Carcass length (cm)	109.00	107.25	107.67	105.00	1.00	0.58
Backfat thickness (mm)	25.11^ab^	26.39^ab^	23.51^b^	27.91^a^	0.62	0.07
Fat percentage (%)	15.67^a^	13.05^b^	12.24^b^	16.65^a^	0.59	< 0.01
Fat-free lean percentage (%)	63.69^b^	64.47^b^	69.57^a^	61.24^b^	0.91	< 0.01
LMA^1^ (cm^2^)	29.16	29.01	31.55	29.23	0.89	0.73

^1^LMA: longissimus dorsi muscle area. ^a,b,c^Values in the same row with different superscript letters are significantly different at *P* < 0.05.

**Table 3:** Serum metabolites levels of finishing pigs with different levels of dietary CGA supplementation.

Items^1^	Control	0.02%	0.04%	0.08%	SEM	*P* value
TP (g/L)	60.60^ab^	52.07^b^	51.66^b^	66.75^a^	1.83	< 0.01
ALB (g/L)	32.74^ab^	32.48^ab^	31.72^b^	36.55^a^	0.74	0.08
GLB (g/L)	24.37^ab^	19.55^b^	19.94^b^	31.33^a^	1.50	< 0.01
UR (mmol/L)	5.67^a^	4.29^b^	4.33^b^	3.79^b^	0.19	< 0.01
CREA (umol/L)	199.34^ab^	195.08^b^	210.24^a^	203.40^ab^	2.23	0.09

^1^TP: total protein; ALB: albumin; GLB: globulin; UR: urea; CREA: creatinine. ^a,b,c^Values in the same row with different superscript letters are significantly different at *P* < 0.05.

**Table 4:** Free amino acids in the serum of finishing pigs with different levels of dietary CGA supplementation.

Items (*μ*g/mL)	Control	0.02%	0.04%	0.08%	SEM	*P* value
Essential amino acids (EAAs)						
Arginine	67.56^b^	214.40^a^	251.43^a^	216.40^a^	17.29	< 0.01
Histidine	33.45^b^	92.06^a^	92.13^a^	97.80^a^	6.81	< 0.01
Isoleucine	51.31^b^	137.69^a^	148.36^a^	126.04^a^	9.31	< 0.01
Leucine	75.89^b^	251.26^a^	268.44^a^	239.34^a^	19.11	< 0.01
Lysine	97.69^b^	284.82^a^	302.17^a^	255.35^a^	21.28	< 0.01
Methionine	15.78^b^	50.84^a^	51.26^a^	46.30^a^	3.78	< 0.01
Phenylalanine	48.57^b^	130.07^a^	137.23^a^	125.71^a^	8.97	< 0.01
Threonine	58.66^b^	189.56^a^	172.29^a^	182.92^a^	13.11	< 0.01
Valine	140.54^b^	447.42^a^	455.37^a^	394.01^a^	34.02	< 0.01
EAAs^1^	562.60^b^	1782.80^a^	1758.70^a^	1683.90^a^	129.54	< 0.01
Nonessential amino acids (NEAAs)						
Alanine	199.55^c^	708.80^ab^	561.97^b^	832.59^a^	61.36	< 0.01
Asparagine	26.15^b^	65.84^a^	83.21^a^	62.09^a^	6.24	< 0.01
Cysteine	6.34^b^	39.20^a^	43.36^a^	47.40^a^	6.02	< 0.01
Glutamine	157.97^b^	291.36^ab^	371.43^a^	428.93^a^	33.40	< 0.01
Glycine	276.10^b^	854.50^a^	937.10^a^	1017.70^a^	74.37	< 0.01
Serine	37.30^b^	117.70^a^	112.07^a^	124.71^a^	9.85	< 0.01
Tyrosine	37.61^b^	107.23^a^	98.47^a^	94.30^a^	6.82	< 0.01
Proline	73.57^b^	217.48^a^	202.59^a^	199.95^a^	14.78	< 0.01
NEAAs^2^	792.91^c^	2307.78^ab^	2145.43^b^	2752.84^a^	176.17	< 0.01
TAAs^3^	1573.82^b^	4090.58^a^	3904.16^a^	4436.72^a^	267.08	< 0.01

^1^EAAs: essential amino acids, including arginine (Arg), histidine (His), isoleucine (Ile), leucine (Leu), lysine (Lys), methionine (Met), phenylalanine (Phe), threonine (Thr), and valine (Val). ^2^NEAAs: nonessential amino acids, including alanine (Ala), asparagine (Asp), cysteine (Cys), glutamine (Glu), glycine (Gly), serine (Ser), tyrosine (Tyr), and proline (Pro). ^3^TAAs: total amino acids. ^a,b,c^Values in the same row with different superscript letters are significantly different at *P* < 0.05.

**Table 5:** Effects of dietary CGA supplementation on free amino acid profile in the LD muscle of finishing pigs.

Items (*μ*g/g)	Control	0.02%	0.04%	0.08%	SEM	*P* value
Essential amino acids (EAAs)						
Arg	21.82	16.61	18.14	20.36	1.07	0.36
His	19.16	17.47	18.57	17.22	0.70	0.78
Ile	16.54	12.51	13.97	15.43	0.78	0.32
Leu	37.23	28.50	30.86	33.76	1.50	0.21
Lys	24.75^b^	23.37^b^	42.73^a^	27.48^b^	2.35	< 0.01
Met	14.24	7.33	9.09	8.57	0.98	0.38
Phe	123.34	114.73	98.68	119.35	4.09	0.13
Thr	96.29	89.93	93.51	95.90	4.44	0.97
Val	24.97^b^	24.21^b^	33.97^a^	29.22^ab^	1.30	0.03
BCAAs^1^	85.03	65.22	69.81	78.41	3.49	0.20
EAAs^2^	409.07	362.62	333.78	368.47	13.09	0.26
Nonessential amino acids (NEAAs)						
Ala	109.36	100.93	99.04	122.08	6.22	0.62
Asp	6.50	5.93	6.76	7.59	0.33	0.34
Cys	8.81^b^	10.67^a^	10.45^a^	9.87^ab^	0.27	0.04
Glu	65.29	59.99	73.50	59.93	4.01	0.58
Gly	43.34	43.37	45.20	42.80	1.40	0.95
Ser	48.52	45.36	46.83	47.83	1.85	0.95
Tyr	15.59^c^	18.79^ab^	20.00^a^	17.21^bc^	0.49	< 0.01
Pro	12.44	11.52	14.41	11.20	0.64	0.25
FAAs^3^	254.64	213.85	234.39	264.32	12.07	0.54
NEAAs^4^	324.32	295.37	303.67	333.25	13.25	0.76
TAAs^5^	733.30	638.59	637.45	701.72	25.18	0.47

^1^BCAAs: branched-chain amino acids, including Ile, Leu, and Val. ^2^EAAs: essential amino acids, including Arg, His, Ile, Leu, Lys, Met, Phe, Thr, and Val. ^3^FAAs: flavor amino acids, including Glu, Asp, Ala, Arg, and Gly. ^4^NEAAs: nonessential amino acids, including Ala, Asp, Cys, Glu, Gly, Ser, Tyr, and Pro. ^5^TAAs: total amino acids. ^a,b,c^Values in the same row with different superscript letters are significantly different at *P* < 0.05.

**Table 6:** Effects of dietary CGA supplementation on free amino acid profile in the BF muscle of finishing pigs.

Items (*μ*g/g)	Control	0.02%	0.04%	0.08%	SEM	*P* value
Essential amino acids (EAAs)						
Arg	29.12	28.79	29.63	30.23	0.90	0.96
His	24.04	23.34	25.05	24.93	0.41	0.45
Ile	20.62^ab^	20.95^ab^	17.53^b^	23.55^a^	0.79	0.04
Leu	46.52	41.74	42.67	47.43	1.00	0.12
Lys	36.85	35.13	30.34	33.47	1.88	0.71
Met	13.60	13.94	12.99	16.23	0.44	0.10
Phe	133.24	127.39	123.11	135.70	3.75	0.65
Thr	178.84^b^	178.13^b^	227.74^a^	140.11^b^	0.34	< 0.01
Val	31.57	35.74	40.61	38.62	1.39	0.13
BCAAs^1^	106.56^ab^	94.56^b^	90.84^b^	113.91^a^	3.30	0.03
EAAs^2^	381.75	337.81	342.51	362.37	8.92	0.30
Nonessential amino acids (NEAAs)						
Ala	163.91	209.80	192.37	201.69	7.93	0.22
Asp	6.80^a^	4.84^b^	5.34^ab^	5.44^ab^	0.28	0.09
Cys	10.34	9.61	10.61	9.76	0.29	0.60
Glu	111.43	102.97	104.27	98.11	3.92	0.70
Gly	68.67^b^	82.28^a^	67.67^b^	75.54^ab^	2.31	0.07
Ser	87.50^b^	82.97^b^	109.88^a^	74.63^c^	3.36	< 0.01
Tyr	20.17	18.28	18.05	18.39	9.73	0.39
Pro	32.55^a^	29.23^a^	32.41^a^	22.95^b^	1.07	< 0.01
FAAs^3^	371.14	418.36	402.58	399.10	8.84	0.31
NEAAs^4^	674.67	662.88	713.99	645.26	13.30	0.29
TAAs^5^	1000.20	1027.16	1056.50	1007.63	17.52	0.68

^1^BCAAs: branched-chain amino acids, including Ile, Leu, and Val. ^2^EAAs: essential amino acids, including Arg, His, Ile, Leu, Lys, Met, Phe, Thr, and Val. ^3^FAAs: flavor amino acids, including Glu, Asp, Ala, Arg, and Gly. ^4^NEAA: nonessential amino acids, including Ala, Asp, Cys, Glu, Gly, Ser, Tyr, and Pro. ^5^TAAs: total amino acids. ^a,b,c^Values in the same row with different superscript letters are significantly different at *P* < 0.05.

## Data Availability

The data used to support the findings of this study are included in the article.

## Supplementary Materials

Supplementary Materials Table S1: composition and nutrient levels of the experimental diets (air-dry basis). Table S2: primers used for real-time PCR analysis.
