# Supplementary material for: Effects of Dietary Chlorogenic Acid Supplementation Derived from Lonicera macranthoides Hand-Mazz on Growth Performance, Free Amino Acid Profile, and Muscle Protein Synthesis in a Finishing Pig Model
Source: Oxid Med Cell Longev. 2022 Mar 12;2022:6316611. doi: 10.1155/2022/6316611 (PMC8934221; doi:10.1155/2022/6316611)
Supplement: Supplementary Materials — Table S1: composition and nutrient levels of the experimental diets (air-dry basis). Table S2: primers used for real-time PCR analysis. [file 6316611.f1.doc]

**Supplemental materials**

Table S1: Composition and nutrient levels of the experimental diets (air-dry basis).

Table S2: Primers used for real-time PCR analysis.

**Table S1: Composition and nutrient levels of the experimental diets (air-dry basis).**

| Items | | Dietary levels of CGA^1^ | | | | |
| --- | --- | --- | --- | --- | --- | --- |
|  |  | 0 (control) | 0.02% | 0.04% | 0.08% |  |
| Ingredient composition, % | | | | | | |
| Corn | 67.00 | | 66.98 | 66.95 | 66.90 |  |
| Soybean meal | 23.76 | | 23.76 | 23.77 | 23.78 |  |
| Wheat bran | 6.00 | | 6.00 | 6.00 | 6.00 |  |
| Soybean oil | 0.88 | | 0.88 | 0.88 | 0.88 |  |
| CGA | 0 | | 0.02 | 0.04 | 0.08 |  |
| Lysine | 0.01 | | 0.01 | 0.01 | 0.01 |  |
| Dicalcium phosphate | 0.50 | | 0.50 | 0.50 | 0.50 |  |
| Limestone | 0.55 | | 0.55 | 0.55 | 0.55 |  |
| Salt | 0.30 | | 0.30 | 0.30 | 0.30 |  |
| 1% premix^2^ | 1.00 | | 1.00 | 1.00 | 1.00 |  |
| Total | 100.00 | | 100.00 | 100.00 | 100.00 |  |
| Nutrient levels^3^, % | | | | | | |
| Digestible energy (MJ/kg) | 14.21 | | 14.21 | 14.20 | 14.20 |  |
| Crude protein^4^ | 16.02 | | 16.00 | 16.01 | 16.02 |  |
| Lysine | 0.73 | | 0.73 | 0.73 | 0.73 |  |
| Methionine + cysteine | 0.51 | | 0.51 | 0.51 | 0.51 |  |
| Threonine | 0.52 | | 0.52 | 0.52 | 0.52 |  |
| Tryptophan | 0.17 | | 0.17 | 0.17 | 0.17 |  |
| Leucine | 1.30 | | 1.30 | 1.30 | 1.30 |  |
| Total calcium^4^ | 0.50 | | 0.50 | 0.51 | 0.49 |  |
| Total phosphorus^4^ | 0.46 | | 0.45 | 0.44 | 0.45 |  |
| Available phosphorus | 0.19 | | 0.19 | 0.19 | 0.19 |  |

^1^ CGA: chlorogenic acid; the same as below. ^2^ Supplied per kg of diet: CuSO_4_·5H_2_O 19.8 mg; KI 0.20 mg; FeSO_4_·7H_2_O 400 mg; NaSeO_3_ 0.56 mg; ZnSO_4_·7H_2_O 359 mg; MnSO_4_·H_2_O 10.2 mg; Vitamin K (menadione) 5 mg; Vitamin B_1_ 2 mg; Vitamin B_2_ 15 mg; Vitamin B_12_ 30 μg; Vitamin A 5,400 IU; Vitamin D_3_ 110 IU; Vitamin E 18 IU; Choline chloride 800 mg; Antioxidants 20 mg; Fungicide 100 mg. ^3^ Calculated values. ^4^ Measured values.

**Table S2: Primers used for real-time PCR analysis.**

| Genes ^#^ | Primers | Sequences (5’-3’) | Size, bp |
| --- | --- | --- | --- |
| SNAT2  (SLC38A2) | Forward | TACTTGGTTCTGCTGGTGTCC | 212 |
|  | Reverse | GTTGTGGGCTGTGTAAAGGTG |  |
| LAT1  (SLC7A5) | Forward | TTTGTTATGCGGAACTGG | 155 |
|  | Reverse | AAAGGTGATGGCAATGAC |  |
| ASCT2  (SLC1A5) | Forward | GGATTCTGGACCGCTGCCTT | 362 |
|  | Reverse | GGCTCCTCCGCTCTTCGTTT |  |
| MuRF1 | Forward | AGCACGAAGACGAGAAAATC | 150 |
|  | Reverse | TGCGGTTACTCAGCTCAGTC |  |
| MAFbx | Forward | CCAGAGAGTCGGCAAGT | 373 |
|  | Reverse | GAGGGTAGCATCGCACAAGT |  |
| MSTN | Forward | GTCCCGTGGATCTGAATG | 293 |
|  | Reverse | TTCCGTCGTAGCGTGATA |  |
| β-actin | Forward | TGCGGGACATCAAGGAGAAG | 216 |
|  | Reverse | AGTTGAAGGTGGTCTCGTGG |  |

^#^ SNAT2, sodium-coupled neutral amino acid transporter 2; LAT1, L-type amino acid transporter 1; SLC1A5, solute carrier family 1 member 5; MuRF1, muscle ring finger 1; MAFbx, muscle atrophy F-box; MSTN, myostatin.
